# Utilization of genetic information for medicines development and equitable benefit sharing

**DOI:** 10.3389/fgene.2023.1085864

**Published:** 2023-06-14

**Authors:** Kotone Matsuyama, Chieko Kurihara, Francis P. Crawley, Sandor Kerpel-Fronius

**Affiliations:** ^1^ Department of Health Policy and Management, Nippon Medical School, Tokyo, Japan; ^2^ Kanagawa Dental University, Yokosuka, Kanagawa, Japan; ^3^ Good Clinical Practice Alliance–Europe (GCPA) and Strategic Initiative for Developing Capacity in Ethical Review, Leuven, Belgium; ^4^ Department of Pharmacology and Pharmacotherapy, Faculty of Medicine, Semmelweis University, Budapest, Hungary

**Keywords:** medicines development, pharmacogenomics, inclusive governance, global public good, global health, benefit sharing

## Abstract

Advances in genomic research have significantly enhanced modern drug development. However, equitable benefit sharing of the results of scientific advancement has not always been achieved. This paper shows how molecular biology has modified medicines development while also leaving open significant challenges for benefit sharing. Presented here is a conceptual modeling describing the processes in genetic-related medicines development and how these are related to specific ethical considerations. The focus is on three important areas: 1) population genetics and the need for discrimination prevention; 2) pharmacogenomics and the need for inclusive governance; and 3) global health to be achieved in open science frameworks. Benefit sharing is taken as the ethical value that underlies all these aspects. The implementation of benefit sharing requires a value shift in which the outcomes of health science are not viewed simply as trade commodities but also as a “global public good”. This approach should lead to genetic science to contribute to promoting the fundamental human right to health to all members of the global community.

## 1 Introduction

Modern drug development underwent rapid and profound changes in the last decades. This is perhaps, especially the case with regard to the role of molecular biology in the development of both medicines and diagnostics (Agarwal et al., 2015), (Schwaederle et al., 2015) which have enabled targeted drug therapy for developing new horizons of patient care. In the last decades new types of medicines, biological agents, and most recently nucleic acid-based drugs enriched and diversified the medicines armamentarium. The precise targeting of several new drugs requires elaborate diagnostic workup, frequently including the demonstration of the presence of specific genomic alterations.

Various human hormones and cytokines produced by recombinant DNA technology are now broadly used for treating many patients who either cannot synthetize these important proteins or need pharmacological amount of these compounds for therapy (Purandarey, 2009). Probably the most important success in the group of biologicals is the development of monoclonal antibodies (mAbs) (Nelson et al., 2010; Viala et al., 2018) and various Fc-fusion proteins (Rath et al., 2015). These technologies open new approaches for the treatment of diseases. Recently the scientific community welcomed the United States (US) Food and Drug Administration’s marketing authorization of the hundredth monoclonal antibody (Mullard, 2021). Their number and structural diversity increase continually opening up new therapeutic pathways. Many mAbs target specific receptors localized on the cell surface where they interfere with the binding of the natural ligands. Following the binding of the specific ligands, the intracellular domains of the receptors are activated. This in turn activates other molecules of the signal transduction pathway, which forward the activation to the cell nucleus. Most of these molecules are specific kinases that, due to constant activation following chromosomal translocation or following their mutations can lead to tumor growth. Many successful drug development programs against tumors aim to inhibit the pathologically active kinases with small molecular weight kinase inhibitors (Ferguson and Gray, 2018; Minnelli et al., 2020). For their effective targeting, the presence of the specifically altered kinase molecule must be demonstrated *in situ*, thus, in the patient. This is most precisely done by sequencing the tumor DNA to identify the sequence coding for the kinase molecule. The knowledge of the human genome and modern technology makes the routine use of DNA sequencing possible for both clinical research and clinical practice (Smedley et al., 2021).

Nucleic acid-based medicines represent a variety of molecular mechanisms (Sridharan and Gogtay, 2016; Kulkarni et al., 2021). Gene therapy aims to replace pathologic genes with normal gene DNA (High and Roncarolo, 2019; Shahryari et al., 2019; Mendell et al., 2017; Naddaf, 2022). The recently developed production method for rapidly synthetizing large quantities of messenger RNA (mRNA) was already successfully applied for vaccine production against COVID-19 infection (Dolgin, 2021; Pascolo, 2021). mRNA therapy can be adapted also for treating rare genetic diseases and for immune therapy in cancer patients (Sahin et al., 2014; Miao et al., 2021; Kiaie et al., 2022). Finally, the synthesis of antisense oligonucleotides (ASOs) against specific mRNA nucleotide sequences permits the silencing of specific genes. ASOs and some recently developed small molecular weight compounds specifically binding to pre-mRNA can be also used to modify the translation of the genetic information by modifying the molecular sites where the large pre-messenger RNAs are spliced into the final mRNA molecules directing the synthesis of the individual proteins. Splicing at altered sites will produce different proteins (Le et al., 2019; Dhuri et al., 2020; Hu et al., 2020; Singh et al., 2020; Crooke et al., 2021; Damase et al., 2021).

While rare hereditary diseases may affect individually only a few patients in society, taken together rare diseases affect large numbers of geographical populations or even subpopulations. Rare diseases are caused usually by genetic alterations that themselves are often inherited. The medicines used for treating rare genetic-related diseases are usually classified as orphan drugs because they are applicable only to a small patient population (Gammie et al., 2015; Attwood et al., 2018; Thomas and Caplan, 2019; Chan et al., 2020). In many respects, the diagnostic workup, especially the characterization of the genomic changes needed for the selection of active drug therapy against hereditary diseases and specific tumors is similar. Tumor types having rare genomic modifications can be also classified as rare diseases. Medicines specifically to address rare tumor types are able to enjoy regulatory orphan drug status for which they receive specific marketing incentives and regulatory benefits (Gammie et al., 2015; Chan et al., 2020). The increased use of molecular pathology in selecting the right drugs for the specific tumors made possible the development of new methodologies for cancer trials in which patients are grouped into treatment arms according to their genomic alterations and not necessarily on the basis of their tumor histology (Park et al., 2019). Thus, developments in molecular biology affect not only our ability to target precisely a wider range of diseases but also increase the precision and speed of medicines development.

From the above short overview, it is evident that modern diagnostics and the precise targeting of new types of medicines are based on the detailed knowledge of the genetic disorder underlying many diseases. Human genomic research for a better understanding of the causes of disease and the selection of the right treatments often requires the analysis of a substantial number of human tissue samples and large sets of respective clinical data. Lack of access to genetic information and linked clinical data may significantly reduce and, thus, one could argue, harm the individual patient and the research community’s ability to improve prophylaxis, diagnostics, and therapeutics for addressing the health needs of future patients. This also extends to the need for the potential future re-use and further analysis of clinical and genetic data obtained from individual participants during previous clinical trials.

Successfully demonstrating the safety and efficacy of new drugs in relation to their activity in specific genomic disorders requires a large investment of time and resources (Brown et al., 2022). The exploratory hypothesis must be validated through stepwise phases of clinical trials, which may not ensure a direct benefit to study participants. In addition, processes of regulatory approval and the manufacturing of specific drugs, and the establishment of supply in the public health system take a significant amount of time. Meanwhile, the expanding sharing of tissue samples containing genetic information and personal data gives rise to various concerns, not only privacy risks but also risks associated with the misuse of such information as well as with the potential for discrimination and/or stigmatization of individuals and populations from whom the samples and/or data have been derived, without being able to provide sufficient benefits outweighing risks. This remains a major ethical concern in the use of genomic information for medicines development and equitable benefit sharing (Simm, 2007). Genetic data may be particularly sensitive because of their ability to precisely identify individuals, and predict risks in relatives (van den Heuvel et al., 2021) and potentially in specific populations or communities (Sharp and Foster, 2000; Otlowski et al., 2012) beyond those who may have given their consent to use or re-use their biological materials and/or data. Therefore, it becomes necessary to evaluate risks beyond individuals to family members and to relevant communities. In this context, the appropriate consent of each study participant, as well as consensus among the relevant population and society, must be sought (Sharp and Foster, 2000).

In connection with the above short outline of advancements in modern medicines development, we will provide an attempt at conceptual modeling to describe the development process of the scientific disciplines, each related to a key concept of ethical considerations: 1) population genetics and discrimination prevention, 2) pharmacogenetics and inclusive governance, which should be led to the achievement of 3) global health together with open science. We shall explore how benefit sharing is a common key concept throughout all these aspects.

## 2 The advancement of medicines development methodologies and their ethical implications

### 2.1 Population genetics and discrimination prevention

Historically, the application of genetic information to improve diagnostics, drug development, and therapy implementation has been significantly driven by the Human Genome Project, which started in the US in 1990 (Green et al., 2015). Following the announcement of the completion of the first draft of the human genome in 2000, another historic milestone was the launch of the Precision Medicine Initiative in the US in 2015 to promote large-scale genome cohort tracking, along with substantial funding including the fund for the development of information technologies needed for privacy protection (Kurihara et al., 2017). With the Human Genome Project, concerns were expressed over the discrimination of individuals and groups based on genetic factors, as well as the expanded commercialization of genetic research results in the disproportionate sharing of benefits among the rich and poor (HUGO Ethics Committee, 2000; Human Genome Organization Ethics Committee, 2000). A major research funding allocation under the theme of the Ethical, Legal, and Social Implications (ELSI) has been continuously promoted in the US (United States National Institutes of Health, 2023), and this concept has spread worldwide. The concerns for patients with familial genetic diseases were expressed as the right to choose whether to undergo pre-diagnosis, i.e., the “right to know”, and even “the right not to know” (Solhdju, 2020).

On the other hand, the availability of the large sets of genetic information of the population in Iceland made possible by deCODE genetics in 1996 created public concerns globally that were then expressed by the national members of the World Medical Association (WMA) (Snaedal, 2018). This led to a 2002 declaration on health databases and biobanks, revised in 2016 as the Declaration of Taipei (DoT) (The World Medical Association, 2016). The company deCODE genetics published some outstanding findings but went bankrupt in 2009 partly because of Lehman Shock (Hirschler, 2009). deCODE was later in 2012 acquired by the American biotech company Amgen (Amgen, 2012). Having had the experience of analyzing the genetic data and medical records of 150,000 family histories in Iceland, the company began in 2019, with the collaboration of a UK-based initiative biobank, named “UK biobank” (deCODE genetics, 2022).

By calling for fundamental ethical principles to be universally agreed upon, the United Nations Educational, Scientific and Cultural Organization (UNESCO) adopted the Universal Declaration on the Human Genome and Human Rights in 1997 (United Nations Educational, Scientific and Cultural Organization, 1997). This declaration recognizes the fundamental unity of members of the human family and their inherent dignity and diversity, addressing the symbolic meaning of the human genome on the shared human heritage. It states that benefits from scientific advances shall be made available to all. Then in 2003, UNESCO adopted (United Nations Educational, Scientific and Cultural Organization, 2003) the International Declaration on Human Genetic Data to help ensure respect for the dignity of persons and the protection of human rights of individuals, in the collection, processing, use, and storage of human genetic and proteomic data, as well as biological samples. These two declarations reflect the development of international agreements, shifting from the primary assurance of fundamental human rights in genetics to practicable guidelines for activities of research and development, considering the values and risks of commercial use. It stipulates the article for “sharing of benefits” providing practicable examples, e.g., access to medical care, developed resulting from genetic research.

Both documents declared the prohibition of discrimination based on genetic information. Laws prohibiting discrimination mainly in insurance coverage and employment have been legislated in some countries but have not yet realized in other countries (Otlowski et al., 2012; Kim et al., 2021). Not only the legal system but also the scientific community’s ethical codes should be enforced. The Springer Nature editors urged in 2022 in a new policy requiring authors, reviewers, and editors to explicitly consider the potential harm to individuals and populations of the scientific papers they publish (Research must do no harm: new guidance addresses all studies relating to people, 2022).

Freedom, equality, and health (World Health Organization, 1946; United Nations, 1948) are agreed upon in the United Nation’s international treaties following World War II. In the development of genetics in the following decades, ethics principles embedded in the codes of conduct of various research communities prohibited genetic discrimination and/or stigmatization. However, the actual implementation of these principles and their legal frameworks for enforcement have not always followed suit. While the ethical instruments may provide a requisite minimum foundation for avoiding harm, they do not ensure it. In addition, benefit sharing, as we find it endorsed more generally in UNESCO’s *Declaration on Bioethics and Human Rights* (United Nations Educational, Scientific and Cultural Organization, 2005), must be promoted equality among those who need it. Due to the complexity and expense of today’s research and development processes in medicines, realizing benefit sharing is ever more challenging. Population genetics does not necessarily directly generate benefits for individuals. It requires sufficient public understanding (Report of a Royal Society ad hoc Group, 1985) as well as safeguarding individuals and communities against all forms of discrimination. In order to truly benefit from epidemiology, it is necessary to develop diagnostic tools and therapeutic products to provide treatment based on accurate diagnoses. This requires the further development of pharmacogenomics, a discipline that integrates diagnosis and treatment, as described in the next Section 2.2. Such development must be destined to promote the health and wellbeing of not only individuals but also relevant communities. Eventually, this appears to be necessary to achieve the goal of global health, as will be described in Section 2.3.

### 2.2 Pharmacogenomics and inclusive governance

The application of genomic science to medicines development is progressing, and international consensus has been reached on methodologies for academic disciplines such as pharmacogenomics and pharmacogenetics (The Council for International Organizations of Medical Sciences, 2005). These sciences require interdisciplinary collaboration (Kerpel-Fronius et al., 2018) and inclusive governance (Organisation for Economic Co-operation and Development, 2020). International guidelines for regulatory approval of drugs have been also provided on definitions of terms of pharmacogenomics/pharmacogenetics (International conference on harmonisation of technical Requirements for registration of pharmaceuticals for human use, 2007), qualification of genomic biomarkers (International conference on harmonisation of technical Requirements for registration of pharmaceuticals for human use, 2010), as well as genomic sampling and management of genomic data (International conference on harmonisation of technical Requirements for registration of pharmaceuticals for human use, 2017). These documents suggest the progress of genetics far beyond the stages of finding pathological mechanisms of diseases, as well as genetic factors related to drug effects. The discipline includes technological and procedural aspects of developing diagnostic tools, searching for drug targets, and resulting in globally approved quality assurance for products utilized in clinical practice worldwide. This quality is required not only for drugs and diagnostic tools but also for health databases or biobanks as one of the research resources.

In recent medicines development, the presence or absence of a specific gene for a disease target molecule in a patient population has been used to determine the susceptibility of the target disease to treatment. In particular, as typified by the development of therapeutic drugs for cancer, the development of targeted drugs is accompanied by companion diagnostics (U.S. Food and Drug Administration, 2018) that use frequently specific genetic information as a biomarker in order to examine therapeutic efficacy. Application of low-cost and convenient gene panel tests (United States National Institutes of Health and National Cancer Institute, 2022) have been expanding, but they did not lead to correspondingly lower-cost treatments.

Utilizing these qualified methods in successful medicines development and patient care, pharmaceutical companies now require in many cases additional biological samples such as blood or surgical specimens from trial participants for genetic biomarker qualification. The collected bio-samples and the relevant data are frequently used for the development of other therapeutic drugs or otherwise are shared with other researchers or companies according to their requests (Taichman et al., 2017). In addition, by using real-world data and artificial intelligence (AI) (Chen et al., 2019), it is now possible to analyze the accumulated medical and genetic information in daily practice, outside the study projects, controlled by academic institutions or companies. This situation suggests that each “research study” as well as the medical records and samples in “ordinary practice” jointly contribute to the formation of “health databases and/or biobanks”, defined by the WMA’s DoT (The World Medical Association, 2016), which complements the Declaration of Helsinki (DoH) (The World Medical Association, 2013). This requires us some conceptual reconstruction of boundaries and overlapping of “research” and “practice” defined in 1979 in the US by the Belmont Report (The national commission for the protection of human subjects of biomedical and behavioral research, 1979), as well as what is “health databases or biobanks”.

The DoT (The World Medical Association, 2016) calls for the establishment of a governance framework for health databases and biobanks, which must be explained to the individuals who are willing to provide consent to multiple uses of their data or biological materials. Especially in the case of genetic databases and biobanks, participants must fully understand possible risks for themselves, family, relatives, and related communities. In addition to privacy protection and discrimination prevention, the DoT includes three important ethical principles.

• Participants have to be informed in advance about “*the procedures for return of results including incidental findings*”.
• *“The interests and rights of the communities concerned, in particular, when vulnerable, must be protected, especially in terms of benefit sharing.”*
• *“Special considerations should be given to the possible exploitation of intellectual property. Protections for ownership of materials, rights, and privileges must be considered and contractually defined before collecting and sharing the material. Intellectual property issues should be addressed in a policy, which covers the rights of all stakeholders and communicates in a transparent manner (Italic texts are verbatim quotations from the WMA's DoT.)”*

Implementation of these principles would be beyond the ethical obligation of an individual researcher. For the implementation of these principles, institutional governance (Council for International Organizations of Medical Sciences, 2023) as well as consensus among stakeholders must be established for the management of incidental findings, intellectual property, ownership, as well as material transfer agreement. These principles suggest that the research community has to consider about “new social contract (United Nations Educational, Scientific and Cultural Organization, 1998; Shafik, 2021)” with populations, especially those who are vulnerable and deserve to benefit from sharing the results of research and development. This must be incorporated into the governance framework of health databases and biobanks to be explained to individuals and the relevant community.

Such inclusiveness has come to be a part of international common regulatory guidance documents (International Council for Harmonisation of Technical Requirements for Pharmaceuticals for Human Use, 2019; International Council for Harmonisation of Technical Requirements for Pharmaceuticals for Human Use, 2021). Ethical considerations including “stakeholder engagement” has come to be explicitly described in these regulatory requirements. Perspectives of regulators and industries focus on refining the study endpoint and outcome to incorporate patient preference, for successful development (Acquadro et al., 2001). However, it should be noted that “benefit sharing” has not been included in these reflections, which should be recognized as an urgent challenge. Stakeholder engagement has been promoted in various areas of scientific development such as genetics (HUGO Ethics Committee, 2000), infectious diseases (UNAIDS, 2011; World Health Organization, 2016) as well as environmental health (UNECE, 2001). Among all, the 2016 revision of the Guidelines for Health-related Research by the Council for International Organizations of Medical Sciences (CIOMS) recommends community engagement from the early stage of research and development in order to ensure product availability in this target community (Council for International Organizations of Medical Sciences, 2016). This requires several structural reformations of internal mechanisms of research and development (Kurihara et al., 2021) to achieve distributive justice of allocation of the risks and benefits among the relevant stakeholders, most importantly, the study target individuals and communities, which could lead to the fairness of benefit sharing.

### 2.3 Global health and open science

Ethics of “patent monopoly” on genome sequence has been discussed since the 1980s, especially in the US, most prominently in the case of the BRCA1/2 patent, which impedes both affordability for patients and scientific progress (Cook-Deegan et al., 2010). The gene patent has raised calls for alleviating the steep rise in the price of genetic testing and maintaining scientists’ motivation for innovation, with a policy not patenting biological information itself while granting the use of derived data. It should be noted that European Union adopted, in order to safeguard the “*dignity and integrity of the person*”, the principle that *“the human body, at any stage in its formation or development, including germ cells, and the simple discovery of one of its elements or one of its products, including the sequence or partial sequence of a human gene, cannot be patented”* (European Communities, 1998). Meanwhile, in some cases, patients and families have become co-holders of the patent (Norrgard, 2008). Between 2000 and 2003, Myriad Genetics was granted several patents in the US and other countries for sequences, mutations, and detection methods for the breast cancer susceptibility genes BRCA1 and BRCA2. Myriad’s patent prohibits other researchers and companies from developing their own, cheaper, and more effective genetic tests for the BRCA gene (Norrgard, 2008).

The issue of access to the benefits resulting from research and obstacles of patent monopoly has also been repeatedly revisited in pandemics due to HIV/AIDS and COVID-19. During the HIV/AIDS pandemic, the “post-trial access” clause in the WMA’s DoH raised substantial debates to avoid the exploitation of research participants in the vulnerable global south. Trial participants and host communities have not been adequately guaranteed the right of access to the developed products. Responding to the HIV/AIDS pandemic, the World Trade Organization (WTO) endorsed Doha Declaration (World Trade Organization, 2001) on prioritizing public health in the Agreement on Trade-Related Aspects of Intellectual Property Rights (TRIPS Agreement).

In the COVID-19 pandemic, immediate public disclosure of the genome sequence of the virus and intensive collaboration among researchers, governments, pharmaceutical companies, patients, and citizens enabled unprecedented rapid, successful vaccine development (Baden et al., 2020; Polack et al., 2020; Voysey et al., 2020), leading to various regional government decisions on mass vaccination. Many scientific papers, including preprints, have been disseminated prior to peer review, and “open science” has been recognized by UNESCO (UNESCO, 2021) to be an urgent issue, with the caution of spreading unvalidated research results or even disinformation, cautioned as “infodemics” (World Health Organization).

To overcome the health crisis which disproportionately affects vulnerable communities in the world, a TRIPS waiver for COVID-19-related products was proposed by South Africa and India (Council for Trade-Related Aspects of Intellectual Property Rights, 2020), resulting in a limited waiver only to the COVID-19 vaccine (World Trade Organization, 2022). Such experience highlighted the challenges of benefit sharing. An international collaboration of industries, governments, and scientists for facilitating technology transfer to develop the capacity of middle-income countries to manufacture products and export to low-income countries. This is the practical transformation of the idea of open science into reality for assuring people’s fundamental rights of access to health. Also, it should be cautioned that fundamental documents for research involving humans such as WMA’s DoH as well as the planned “renovation” of ICH-GCP(R3) (ICH, 2021), benefit sharing has not been well assured.

The COVID-19 crisis reminded the world of the evils of patent monopoly and the importance of solving globally common challenges through solidarity. This consideration should not be limited to the cases of a major pandemic. The right to health must be ensured also for marginalized small-size populations with rare genetic diseases so that no one is left behind, as designated by the United Nations to be the Sustainable Development Goals (SDGs) (United Nations, 2015). To this end, benefit sharing must be included as a key element of the operating mechanism in all areas of scientific disciplines as well as research and development leading to all processes of product lifecycle management.

## 4 Discussion

We analyzed how recent advances in genetic medicine in terms of population genetics and pharmacogenomics, progressing towards the achievement of global health, have been transforming the medicines development landscape. For each process, “discrimination prevention”, “inclusive governance” and “open science” have been presented as key concepts, while benefit sharing is the key ethical value underlying all of them. This process may imply that the focus of ethical concerns has been shifting from “non-maleficence” to more promoting “beneficence”.

Genetic science has been expected to overcome intractable diseases and promote precision medicine. Precision medicine is not developed only for wealthy populations, resulting in a higher price of targeted drug therapy, leading to expanding health disparities due to social determinants of health (Yedjou et al., 2019). On the contrary, it must be promoted to achieve global public health, to be assured for the people who most need the achievement of science. To resolve the dilemma of “tragedies of commons (The Tragedy of the Commons, 1968) and anti-commons (Heller, 2023),” reformation of the set of values is needed.

This paper focuses mainly on ethical issues in managing a substantial volume of genetic data, which is especially sensitive private information. Ethical considerations of gene therapy and gene editing are not discussed, because these interventions require different aspects. The ethical and social issues of exorbitantly high prices of several nucleic acid-derived drugs including the possibility of benefit sharing were already discussed in a previous paper (Kerpel-Fronius et al., 2020). Also, somatic, germline, and heritable genome editing raised substantial debate related to the technological modification of human identity, threatening human dignity, and now procedural recommendations for governance have come to be given (World Health Organization, 2021). These issues require further consideration from the perspective of benefit sharing.

## 5 Conclusion

As technology becomes more sophisticated, research and development require a larger volume of personal data and biological materials in health databases and biobanks, sometimes repeatedly. In such an environment, benefit sharing must be ensured by involving relevant communities from the early stages of a project of research and development, as well as the establishment of health databases and biobanks. In addition, in the process of informed consent of each participant, not only an acceptable profile of direct and prospective risks and benefits but also a practicable plan for ensuring benefit sharing must be described. In such a landscape, information derived from research involving humans and health products achieved from research should not be regarded as a commercial commodity, but a “global public good” (World Health Organization, 2002) equally shared among the global community for assurance of health and wellbeing, as fundamental human rights.

## Data Availability

The original contributions presented in the study are included in the article, further inquiries can be directed to the corresponding author.
